# Social Correlates in Reproductive Tract Infections among Married Women in Rural Area of Meerut

**DOI:** 10.4103/0970-0218.39246

**Published:** 2008-01

**Authors:** Bhawna Pant, JV Singh, M Bhatnagar, SK Garg, H Chopra, SK Bajpai

**Affiliations:** Department of Community Medicine, Subharati Medical College, Meerut, India; 1 Department of Community Medicine, LLRM Medical College, Meerut, India

## Introduction

NFHS-2 (1998-1999) results show that more than one-third of ever married women in India report at least one reproductive health problem related to vaginal discharge or urination; and two-fifths of currently married women report at least one reproductive health problem related to vaginal discharge, urination or intercourse that could be a symptom of more serious reproductive tract infection.(1) Majority of women bear the problems silently without seeking advice and treatment.(2,3)

The present study was undertaken to assess the magnitude of the problem of RTIs and its social correlates among married females aged 15-44 years in rural Meerut.

## Materials and Methods

The study was cross-sectional in design. The sample size of 470 was calculated assuming the RTI prevalence of 45% with relative precision of 10% at 95% confidence level. But to overcome the noncooperation, non-availability and refusal of internal examination, a total of 600 married women aged 15-45 years were sought to be covered. The study was conducted in 12 randomly selected villages out of 24 sub centre villages under the field practice area of Department of SPM Medical College, Meerut.

They were interviewed through house-to-house survey by a lady doctor. The desired information was collected on a pre-designed and pre-tested interview schedule.

Consent was duly taken.

## Results

Overall, 600 women (15-45 years) were recruited for the study. Many (212, 35.3%) women reported symptoms suggestive of reproductive tract infection.

Table 1 shows the relationship between social correlates and RTI.

**Table 1:** Relationship of reproductive tract infections with socio-demographic and reproductive health variables

Socio-demographic and reproductive health variable	Total surveyed	RTI cases		χ^2^	*P* value
				
		No.	Prevalence (%)		
Age-wise distribution					
15-19	41	15	36.5	14.17	<0.02
20-24	71	21	29.5		
25-29	150	69	46		
30-34	123	36	29.2		
35-39	139	39	28		
40-44	76	32	42.1		
Type of family					
Nuclear	340	103	30.2	8.7	<0.05
Joint	260	109	41.9		
Women's education					
No schooling	448	167	37.3	9.13	<0.05
1^st^-8^th^ class	80	31	38.8		
9^th^ and above	72	14	19.4		
Age at marriage					
Less than 18 years	208	85	40.9	4.28	<0.05
More than or equal to 18 years	392	127	32.4		
Personal hygiene					
Good	227	57	25.1	17.1	<0.001
Fair	329	135	41		
Poor	44	20	45.4		
Menstrual hygiene					
Use dirty clothes	417	168	40.2	14.7	<0.001
Use the clothes after washing + Sanitary pads	183	44	24.04		
Family planning methods					
Non-users	414	419	36	9.69	<0.001
Users	186	63	33.80		
Condoms	37	9	24.30		
Pills*	30	4	13.30		
IUD*	9	3	33.30		
Sterilization	110	47	42.70		
Total	600	212	35.3

* Pooled together for χ^2^

Prevalence of RTI was maximum (46%) in the 25-29 years age group, followed by 40-44 years (42.1%). The prevalence of RTI was significantly higher (41.9%) in women belonging to joint families as compared to prevalence in women belonging to nuclear families (30.2%) (*P* < 0.005).

Majority (74.7%) of married women had no schooling. The proportion of women educated up to classes I to VIII and women educated up to above class VIII was 13.3% and 12% respectively. The prevalence of RTI was higher in illiterate and less educated (up to class VIII) women (37.3% and 38.8% respectively) as compared to 19.4% in more educated women (*P* < 0.05).

One-third of the study population was married before the age of 18 years. The prevalence of RTI was significantly higher (40.9%) in women who were married at an age below 18 years as compared to 32.4% in women who got married at an age above 18 years (χ^2^ = 4.28, d.f. 1, *P* < 0.05).

Prevalence of RTI was 45.4%, 41% and 25% in women having poor, fair and good personal hygiene respectively. The prevalence of RTI was significantly higher (*P* < 0.001) in women who used unworked clothes during menstruation (40.2%) as compared to women who used either washed clothes (23.7%) or sanitary pads (28.6%) (*P* < 0.001).

Prevalence of RTI was maximum (42.7%) in women who had adopted sterilization and minimum (13.3%) among the oral pill users (*P* < 0.02).

## Discussion

A rather high prevalence of RTI (35%) in married females of age 15-44 years was reported in our study. This is similar to the prevalence reported by Nandan *et al.* in rural Agra (34%) and higher than 29% reported by Singh.(3)

Age-specific prevalence of RTI as reported in our study being maximum in women aged 25-29 years (46%) followed by 36.5% in women aged 15-19 years was at variance with the reports by others - for example, Nandan *et al.*(4) reported a maximum prevalence of 60.86% in the younger age group (15-24 years), while Rathor *et al.*(5) reported a maximum prevalence of 44.7% in the age group 40-44 years.

Higher prevalence of RTI among women with lower literacy and poor hygiene indicates a role of life style and personal hygiene in the causation of RTI. Significantly higher prevalence of RTI (40.9%) in women who were married at an age below 18 years indicates that early sex and pregnancy are unhealthful for girls in every way, lengthening the span of years over which they have children and increasing the risk of infections.

In our study, the prevalence of RTIs was significantly low in those women who were using barrier methods (24.3%). Barrier contraceptives are known to provide protection against RTIs/ STIs; the prevalence of RTIs was more in IUD users (33.3%) and those using terminal methods of contraception (42.7%). This finding has obvious implication for family planning program.

*Limitation of the study:* Treatment-seeking behavior was not explored.
